# Strategies for Drug Delivery into the Brain: A Review on Adenosine Receptors Modulation for Central Nervous System Diseases Therapy

**DOI:** 10.3390/pharmaceutics15102441

**Published:** 2023-10-10

**Authors:** Mercedes Fernandez, Manuela Nigro, Alessia Travagli, Silvia Pasquini, Fabrizio Vincenzi, Katia Varani, Pier Andrea Borea, Stefania Merighi, Stefania Gessi

**Affiliations:** 1 Department of Translational Medicine, University of Ferrara, 44121 Ferrara, Italy; mercedes.fernandez@unife.it (M.F.); manuela.nigro@unife.it (M.N.); trvlss@unife.it (A.T.); fabrizio.vincenzi@unife.it (F.V.); vrk@unife.it (K.V.); 2 Department of Chemical, Pharmaceutical and Agricultural Science, University of Ferrara, 44121 Ferrara, Italy; silvia.pasquini@unife.it; 3 University of Ferrara, 44121 Ferrara, Italy; bpa@unife.it

**Keywords:** blood–brain barrier, brain microvascular endothelial cells, tight junction proteins, adenosine receptor signaling, adenosine receptor agonists, adenosine receptor antagonist

## Abstract

The blood–brain barrier (BBB) is a biological barrier that protects the central nervous system (CNS) by ensuring an appropriate microenvironment. Brain microvascular endothelial cells (ECs) control the passage of molecules from blood to brain tissue and regulate their concentration-versus-time profiles to guarantee proper neuronal activity, angiogenesis and neurogenesis, as well as to prevent the entry of immune cells into the brain. However, the BBB also restricts the penetration of drugs, thus presenting a challenge in the development of therapeutics for CNS diseases. On the other hand, adenosine, an endogenous purine-based nucleoside that is expressed in most body tissues, regulates different body functions by acting through its G-protein-coupled receptors (A1, A2A, A2B and A3). Adenosine receptors (ARs) are thus considered potential drug targets for treating different metabolic, inflammatory and neurological diseases. In the CNS, A1 and A2A are expressed by astrocytes, oligodendrocytes, neurons, immune cells and ECs. Moreover, adenosine, by acting locally through its receptors A1 and/or A2A, may modulate BBB permeability, and this effect is potentiated when both receptors are simultaneously activated. This review showcases in vivo and in vitro evidence supporting AR signaling as a candidate for modifying endothelial barrier permeability in the treatment of CNS disorders.

## 1. Introduction

Current trends in applied and translational neuropharmacology involve the development of new drugs for the treatment of specific central nervous system (CNS) diseases, including the development of strategies that can overcome the blood–brain barrier (BBB) and enable drugs to reach the brain’s microenvironment by directing them to the specific/target cells involved in such diseases [1,2].

Compared to other therapeutic fields, drug discovery for brain diseases is very unsuccessful mainly due to the high complexity of the brain, the long time needed for drug development, the lack of effective approaches for drug delivery into the brain, and significant drug side effects. Furthermore, due to the BBB’s limited permeability, only around 2% of small-molecule drugs can cross the BBB on the basis of their molecular size, charge and hydrophobicity, as well as multidrug resistant protein substrate activity [1,3,4,5,6,7,8].

Although BBB is the major obstacle for delivering drugs into the CNS, it plays a critical role in the brain’s metabolic activity, as well as neuronal function. Under healthy conditions, the brain is constantly and efficiently supplied of oxygen, nutrients and essential amino acids, while neurotoxic substances are taken away to maintain its homeostatic balance [9].

The BBB also plays a pivotal role in the regulation of some neurotransmitters’ trafficking between the peripheral and central nervous systems for which a specific transport system across the BBB exists. Such is the case for adenosine, a purine nucleoside that acts through purinergic P1 receptors that are selective for adenosine [10,11]. It functions as an important local signaling molecule and is involved in various physiological functions, including neurotransmission, cardiac pace and immune regulation [12,13]. Adenosine, produced both intracellularly and extracellularly, mediates its function by acting through its 7-transmembrane G-protein-coupled receptors, A1, A2A, A2B and A3 adenosine receptors (A1R, A2AR, A2BR and A3R), which are widely distributed on various cell types in the body [14,15,16,17]. Among them, BBB microvascular endothelial cells (ECs) express A1Rs and A2ARs.

On the other hand, it has been described that selective agonists of A1R and A2AR induce a transient increase in the BBB permeability, thus allowing the entry of macromolecules into the CNS. This effect is, in turn, counteracted by blocking adenosine signaling through selective ARs antagonists [18], which suggests that adenosine signaling might be considered a strategy to modify the BBB permeability.

This review focuses on purinergic signaling mediated by P1 ARs as a putative tool to modify endothelial barrier permeability to facilitate the flow of substances across the BBB. In this paper, we discuss recent in vivo and in vitro research performed to understand how the BBB can be modified in some brain pathological conditions and exploit the ARs signaling through the use of different types of AR agonists and antagonists. An overview of current state of the art of new strategies that can be applied to increase BBB permeability to enable the delivery of therapeutic drugs for the treatment of CNS diseases is provided.

## 2. Biological Brain Barriers and Brain Drug Delivery

**Figure 1:**
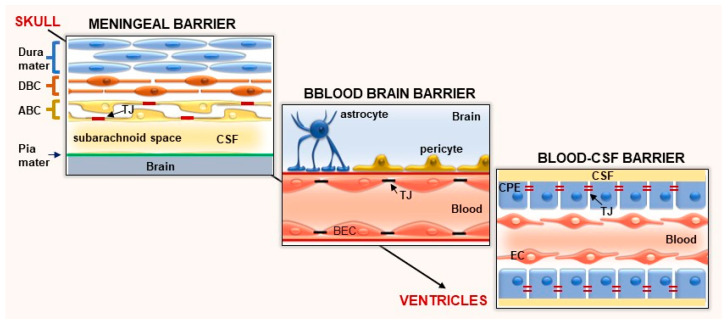
Biological barriers of the CNS. The meningeal barrier possesses the most complex structure of all the brain barriers. It is made of arachnoid barrier cells (ABC) and tight junctions (TJs) between adjacent cells forming a barrier between the cerebrospinal fluid (CSF) in the subarachnoid space (SAS) and the dural border cells (DBC) and the dura mater. The blood–brain barrier is made of brain endothelial cells (BECs), inter-endothelial junctions (TJs, adherens junctions and gap junctions), pericytes, astrocytes and the basal membrane. The blood–CSF barrier is situated in the choroid plexus within each brain ventricle. The barrier is made of cells of the choroid plexus epithelium (CPE) and tight junctions.

As is well known, biological barriers that protect the CNS providing an appropriate microenvironment are: the blood–brain barrier (BBB) constituted by brain microvessel endothelial cells; the blood–cerebrospinal fluid barrier (BCSFB) or blood CSF barrier, formed by choroid plexus epithelial cells; and the meningeal barrier, formed by arachnoid epithelial cells [4] (Figure 1).

These barriers are responsible for controlling the trafficking of molecules to guarantee the function of neuronal circuits, synaptic transmission, angiogenesis and neurogenesis. Moreover, they prevent the entry of immune cells into the brain [4].

The BBB regulates the interaction between blood and the brain through an extended surface of capillaries involved in regulating the passage of molecules/drugs from blood to brain tissue and the concentration-versus-time profiles, i.e., pharmacokinetics (PK). At the same time, PK depends on drug physicochemical properties and, consequently, on its transport across the BBB, as well as on the physiological characteristics of the CNS, including the effect of aging [19,20].

The BBB prevents bloodborne and exogenous molecules from entering the central nervous system and is crucial to maintaining brain homeostasis. The differentiation of the endothelium into a barrier layer begins during embryonic angiogenesis and, in adults, is maintained by a very refined and complex structure made of (i) microvascular endothelial cells (ECs) that delineate the cerebral capillaries in the brain and the spinal cord, providing CNS with a highly complex vasculature; (ii) inter-endothelial junctions that are protein complexes of three types, tight junctions (TJs), adherens junctions and gap junctions; and (iii) pericytes (PCs), astrocytes and the basal membrane (BM), a thin extracellular matrix layer that surrounds ECs, thus giving the BBB its “impermeable” property [4].

ECs possess specific markers including gamma-glutamyl transpeptidase (GGTP), alkaline phosphatase, von Willebrand factor (vWf), glucose transporter-1 (GLUT-1), endothelial barrier antigen (EBA) and OX-47 antigen. Furthermore, ECs are also characterized by the presence of P-glycoprotein (Pgp) and multidrug resistance-associated protein (MRP), which are two N-glycosylated phosphoproteins [21,22].

**Figure 2:**
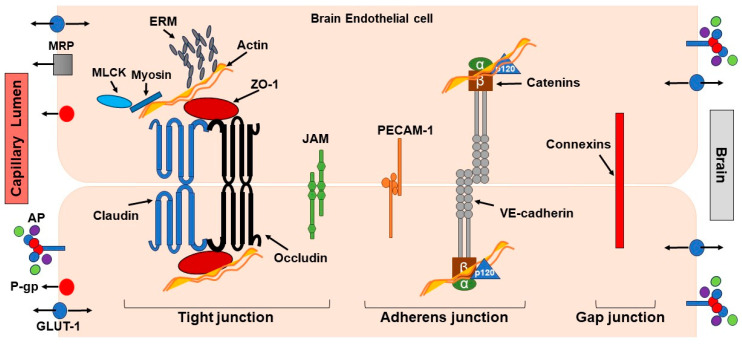
Brain microvascular endothelial cells (ECs)—inter-endothelial junctions complex of the BBB. The high vasculature that ECs provide to the CNS is made of inter-endothelial junctions that link the ECs in a coordinated way forming a continuous barrier. BBB permeability and the communication between ECs are regulated in response to physiological and pathophysiological conditions. Some of the ECs specific markers included in the cartoon are: Alkaline-phosphatase (AP) localized in the plasma membrane and highly expressed in ECs; the macromolecule transporter glucose transporter-1 (GLUT-1); and the two efflux transporter proteins P-glycoprotein (Pgp) and multidrug resistance-associated protein (MRP). There are three types of inter-endothelial junctions: tight junctions (TJs), adherens junctions and gap junctions. TJs consist of interconnected strands of the trans-membrane proteins occludin and claudins (Cln), which form the seal between endothelial cells. These proteins link with the actin cytoskeleton via the accessory proteins ZO-1, a family of cytoplasmatic proteins with regulatory and structural roles in the organization of TJs. Junction adhesion molecules (JAMs) are an immunoglobulin superfamily highly enriched at TJs that contribute to their properties. Ezrin/radixin/moesin (ERM) and myosin light chain (MLC) are actin-binding proteins present at TJs. The phosphorylation of ERM and the phosphorylation of MLC by the MLC kinase (MLCK) provokes the modulation of actin and actomyosin arrangement thus modifying the BBB permeability. Adherens junctions are characterized for the interaction between vascular endothelial (VE)-cadherin and catenin proteins (p120, β- and α-catenin). The platelet endothelial cell adhesion molecule-1 (PECAM-1) is a transmembrane protein that is also present at the adherens junctions and contributes to the regulation of vascular integrity. Together, these proteins control the formation, maintenance and function of adherens junctions. Gap junctions are composed of connexin protein subunits.

The permeability of the BBB is mainly controlled by inter-endothelial junctions, which link ECs in a coordinated way to form a continuous barrier capable of regulating its permeability and the communication between ECs in response to physiological and pathophysiological changes in the brain [9,23] (Figure 2).

Tight junction (TJ) complexes between brain capillary endothelial cells are important structural components of the BBB and consist of interconnected strands of transmembrane proteins and accessory proteins [3]. TJs possess associated transmembrane proteins, occludin and claudin (Cln), involved in the regulation of BBB integrity [24,25]. They act as junctional adhesion molecules that seal endothelial cells together, thus regulating the diffusion of fluid and small molecules across the BBB. Both proteins are essential for BBB formation. It has been reported that an increase in claudin-5 expression is associated with an increase in transendothelial electrical resistance (TEER), a functional parameter of paracellular permeability, and consequently a decrease in BBB permeability [26]. In the context of brain drug delivery, TEER values are indicators of cellular barrier integrity that should be considered when evaluating the transport of drugs into the brain. A decrease in TEER values correlates with increased paracellular space between adjacent endothelial cells and, hence, with increased permeability [27]. Accessory proteins, such as the zona occludens-1 (ZO-1) family, are cytoplasmatic proteins with regulatory roles and are involved in TJ structural organization through the linking of TJ complexes to the cytoskeleton, as well as in the support of protein signal transduction [28,29]. Ezrin/radixin/moesin (ERM) and myosin light chain (MLC) are actin-binding proteins present at TJs. Phosphorylated ERM proteins are involved in actin remodeling and vascular permeability control [30]. In addition, ERM proteins have been found to form adhesion complexes and actin–myosin stress fibers after the activation of Rho GTPases. Moreover, TJ barrier function can be regulated by signal transduction cascades that require the activation of Rho proteins and depend on the actin cytoskeleton. Furthermore, the phosphorylation of MLC by myosin light chain kinase (MLCK) provokes the activation of actomyosin contraction and the increased phosphorylation of MLC corresponds to increased BBB permeability [31,32,33].

Adherens junctions possess multiple functions, including stabilization of cell–cell adhesion, regulation of the actin cytoskeleton, and intracellular signaling and transcription. The interaction between endothelial (E)-cadherin and the p120-, β- and α-catenin families of proteins occurs at adherens junctions [34]. Platelet endothelial cell adhesion molecule-1 (PECAM-1, or CD31) is a transmembrane protein that is also present at adherens junctions and contributes to the regulation of vascular integrity [35]. Connexins constitute a large family of transmembrane proteins that allow intercellular communication and the transfer of ions and small signaling molecules between cells.

Gap junctions are composed of connexin protein subunits. These junctions form channels between two adjacent cells, thus allowing direct intercellular communication [36].

Moreover, the structure that is made of ECs, the capillary BM, astrocytes, pericytes (PCs) embedded within the BM, and microglial and neuronal cells is known as the neurovascular unit (NVU) [4]. The maintenance of barrier function is guaranteed due to the high vascularity of the brain, which allows oxygen and nutrient supply from the peripheral blood to the brain [12,37].

**Figure 3:**
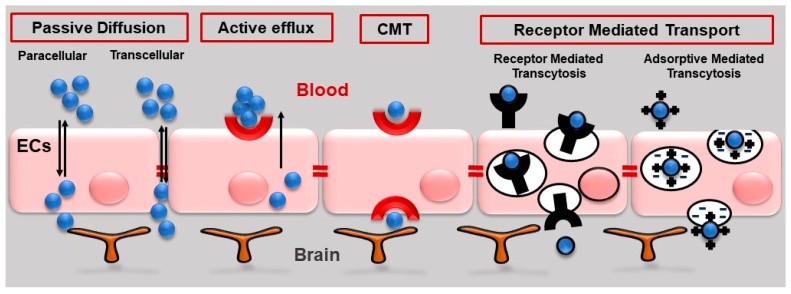
Types of transport across the BBB. Passive diffusion (paracellular and transcellular transport), active efflux, carrier-mediated transport (CMT) and receptor-mediated transport (transcytosis and adsorptive mediated transcytosis).

Different types of transport across the BBB have been described, among them, passive diffusion, active efflux, carrier-mediated transport (CMT) and receptor-mediated transport (Figure 3).

Passive diffusion consists of the passage of the molecules across the BBB by one of the following ways: (i) through the cells, i.e., transcellular transport; and (ii) between adjacent cells, i.e., paracellular transport [38]. In passive diffusion, lipid-soluble small molecules can diffuse passively across the BBB into the brain on the basis of their molecular parameters, such as lipid solubility and molecular weight (lower than 400 Da). As mentioned above, only 2% of small molecules can cross the BBB and only 5% of available drugs can be used to treat CNS diseases [3].

Active efflux is driven by different primary barrier interface proteins. Adenosine triphosphate (ATP)-binding cassette (ABC) transporters are BBB transporter proteins expressed on the luminal endothelial plasma membrane and limit the BBB permeability to toxins and therapeutic compounds. Their expression and functional activity have been found to decrease in neuropathological conditions such as Alzheimer’s disease (AD) and Parkinson’s disease (PD) [39]. Other efflux transporter proteins are P-glycoprotein (Pgp), multidrug resistance-associated proteins (MRPs) and breast cancer resistance protein (BCRP). Pgp and BCRP are expressed in the luminal membrane of the BBB and are responsible for the transport of substrates from the endothelium to blood, whereas MRPs are expressed in both luminal and abluminal membranes. When the primary barrier is dysfunctional, transport activity can be performed by pericytes and perivascular astrocytic endfeet.

Carrier-mediated transport (CMT) is an energy-dependent pathway generally used by small hydrophilic molecules. This type of transport is performed by carriers that possess specific receptors on their membrane that recognize the target molecules (amino acids, carbohydrates, monocarboxylic acids, fatty acids, hormones, nucleotides, organic anions, amines, choline and vitamins) and transport them across the cell membrane [40]. As an example, essential omega-3 fatty acids, such as docosahexaenoic acid (DHA), are transported into the brain via endothelial major facilitator superfamily (MSF) domain-containing protein 2a (MFSD2a) [41], a member of the MFS membrane carrier transporters known to play a critical role in maintaining the integrity of the BBB [42].

Receptor-mediated transport is a non-specific transcytotic mechanism to transport a variety of large bloodborne molecules and complexes across the BBB. In particular, it is used by specific neuroactive peptides, regulatory proteins, hormones and growth factors to cross the BBB. There are two types of receptor-mediated transport: receptor-mediated transcytosis (RMT) and adsorptive-mediated transcytosis (AMT) [43].

On the other hand, in the CNS there is a low infiltration of neutrophils into the brain and the interaction between BBB and immune cells is strictly regulated. It occurs at the endothelial cell junction through paracellular or transcellular transport and is known as diapedesis [44]. Moreover, during embryonic development and under physiological conditions, mononuclear cells enter the brain and become microglia, the resident immunological competent cells of the CNS [45].

On the other hand, different physiological functions have been ascribed to the BBB at the blood–brain interface:

(1) Regulate levels of neurotransmitters present in the central and peripheral nervous systems by keeping these different neurotransmitters’ pools separate to minimize “crosstalk” and protect the brain from sudden changes in plasma levels. This is the case of the neuroexcitatory amino acid glutamate, whose levels vary significantly under specific physiological and/or pathological conditions (food intake and ischemic stroke). High levels of glutamate inside the brain significantly damage neuronal tissue [4].
(2) Maintain ionic homeostasis and brain nutrition via the action of specific ion channels and transporters, which guarantees the optimal conditions for neural and synaptic signaling functions. On the other hand, the BBB, together with the BCSFB, regulates pH levels and also calcium and magnesium homeostasis, which is essential to control neuronal excitability and the transmigration of macrophages across the BBB. Moreover, Ca^2+^ is involved in the modulation of BBB integrity and endothelial morphology [4].
(3) Protect the brain against increased access of neurotoxins of an endogenous and/or exogenous origin by regulating the entry of blood-circulating substances through the ABC energy-dependent efflux transporters (ATP-binding cassette transporters) present in the BBB luminal surface [46].
(4) Limit the outflow of plasma macromolecules into the brain under physiological conditions, thus preventing several pathological conditions that would be provoked when large-molecular-weight serum proteins cross the BBB [4].

Nevertheless, the BBB’s integrity and physiological functions can be altered during pathological conditions. BBB dysfunction can be associated with either moderate and reversible changes due to TJ aperture or with severe and permanent changes in proteins, enzymes and other transport systems [4,47]. Therefore, microglial activation and infiltration of immune cells into the brain occur, provoking an alteration in CNS homeostasis and resulting in possible damage to the brain and thereby causing or exacerbating a pathological condition. The relative impermeable healthy BBB becomes more permeable in response to cytokines, vasoactive agents (for instance, adenosine) and chemical mediators (e.g., ATP, aspartate, nitric oxide and glutamate) that are released during the development of a pathological condition [4].

The blood–cerebrospinal fluid barrier (BCSFB) is formed by the epithelial cells of the choroid plexus (CPE) connected through TJs. These cells secrete CSF into the ventricular brain system in a controlled way [48] (Figure 1).

The meningeal barrier consists of an avascular arachnoid epithelium made of arachnoid barrier cells (ABC) and tight junctions (TJs). Situated under the dura mater, it wraps the CNS and keeps separate the extracellular fluids of the central nervous system and the fluids of the rest of the body. Due to its avascular nature and small surface area, the meningeal barrier contributes marginally to the exchange of substances between blood and the brain [4,49] (Figure 1).

## 3. Brain Drug Delivery Strategies

The BBB constitutes a physical semipermeable and dynamic interface between the blood and the brain and is mainly responsible for managing the exchanges between blood and brain tissue by allowing only certain molecules or ions to pass through via diffusion or via more specialized processes of facilitated diffusion, passive transport or active transport.

Keeping in mind the BBB’s highly organized close-fitting structure, it is easy to understand why it also represents an obstacle for drug delivery to the brain. Thus, knowledge of the molecular and physiological mechanisms involved in the transport of compounds through the BBB represents an important key for CNS drug delivery, and further studies on brain drug pharmacokinetics and distribution into specific areas of the brain are needed [50]. The concept is to modify drugs that cannot cross the BBB in their original state by transporting them through the use of already-identified endogenous BBB carrier-mediated and receptor-mediated transport systems.

In the last forty years, a lot of new technologies aiming to modify the physicochemical properties of drugs to cross the BBB and deliver drugs into the brain have been developed, including BBB disruption, CSF delivery, trans-cranial delivery, lipid carriers, prodrugs, stem cells, exosomes, nanoparticles (NPs), gene therapy, recombinant proteins and nucleic-acid therapies [51,52].

One of the many classifications that can be found through the existing literature groups these brain drug delivery strategies into three categories, and the strategies included in these three categories can be invasive or non-invasive or miscellaneous for the brain depending on their method of action [51].

BBB disruption (BBBD) is a brain drug delivery strategy that can be invasive or miscellaneous. This technique consists of the use of substances that induce modifications of ECs and TJs, thus allowing the passage of certain molecules into the brain. The main disadvantage of this technique is that some substances (e.g., mannitol, glycerol and ethanol) can compromise BBB integrity and physiological functions; thus, noxious blood components that reach the brain tissue can cause injury [50,51,53].

CSF delivery strategies through intracerebroventricular or intrathecal infusion of drugs are invasive brain drug delivery strategies consisting of the injection of therapeutic proteins directly into the CSF. The advantage of these methods is the lower drug concentration of therapeutic drugs compared with regular intravenous administration, while also avoiding the risk of toxicity due to systemic exposure. Intrathecal drug administration can be performed via lumbar puncture or via an implanted intrathecal drug delivery device (IDDD) [54].

Trans-vascular brain drug delivery includes receptor-, carrier- and lipid-mediated transport and active efflux. In this context, transcytosis mediated by receptors (RMT) seems to be the easier way to bypass the BBB by allowing the drug being used to be first taken up via EC receptor-mediated endocytosis (RME), and then the targeting ligand binds to a receptor on the surface of ECs to transport the drug to the abluminal surface of the brain parenchyma via transcytosis [53]. The most commonly used targets in preclinical and clinical studies are transferrin, insulin, low-density lipoprotein, and diphtheria toxin receptors [55,56,57,58,59].

Nanotechnology applied to drug delivery into the brain is an approach mainly based on the use of nanosized technology for drug release in the brain. This delivery system uses a wide variety of nanoscale drug delivery platforms, mainly lipid- and polymer-based NPs, that assure a controlled and improved release of their cargo by protecting the loaded drugs from being metabolized [55]. Current efforts have mainly focused on increasing the ability of NPs to target their therapeutic site, thereby minimizing the doses of drugs released at undesired sites, to enhance the efficiency and, thus, the cost of these therapies [54,60].

Niosomes are a type of vesicular nanocarrier whose non-ionic, surfactant-based and non-toxic characteristics, together with their amphiphilic nature that is compatible for the encapsulation of lipophilic and hydrophilic molecules, make them a new delivery system for active drugs [61]. Many examples of NP applications have been reviewed in detail [4,51,53,60].

## 4. Adenosine, Adenosine Receptors and BBB Modulation: Implications in CNS Homeostasis in Both Physiological and Pathological Conditions

### 4.1. Physiological Conditions

Adenosine is an endogenous purine nucleoside that, under physiological conditions, is mainly generated inside cells from the substrate S-Adenosyl-L-homocysteine through the catalytic action of the enzyme S-Adenosyl-L-homocysteine hydrolase, leading to adenosine and homocysteine as the products. Adenosine is a basic constituent of nucleic acids and can also be present in the forms of AMP, ADP and ATP; these purine molecules play a crucial role in cellular energy supply and in the transfer of information within and across cells [20,62,63].

**Figure 4:**
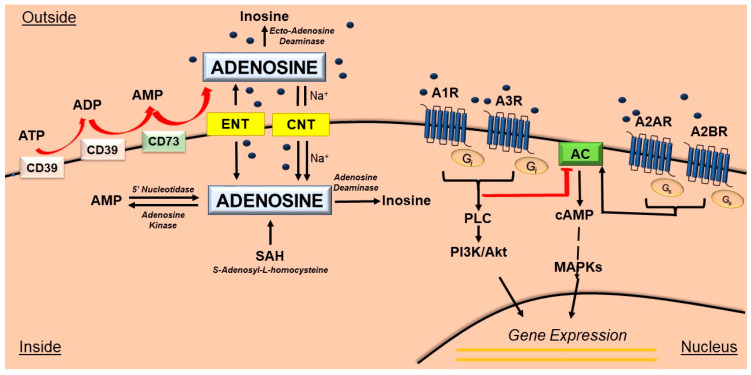
Adenosine metabolism and signal transduction. The nucleoside adenosine is generated from ATP and converted to ADP and AMP by ectonucleoside triphosphate diphosphohydrolase 1 (CD39) followed by conversion to adenosine by ecto-5-nucleotidase (CD73). Inside the cell, the generation of adenosine can occur either from ATP, ADP and AMP via the activity of cytoplasmic 5′-nucleotidases, or from the hydrolysis of S-Adenosyl-L-homocysteine (SAH) via the action of the enzyme S-Adenosyl-L-homocysteine hydrolase. Adenosine is then transported and metabolized to either inosine or AMP by the enzymes adenosine deaminase (AD) or adenosine kinase, respectively. Extracellular adenosine can be metabolized to inosine via the ecto-adenosine deaminase and/or uptaken by either the nucleoside transporters system (ENT) or the concentrative nucleoside transporters system CNT, a sodium-dependent Na+ transporter. Extracellular adenosine activates the adenosine receptors A1, A2A, A2B and A3. A1R and A3 are coupled to an inhibitory G protein (Gi). A2A and A2B are coupled to the stimulatory G protein (Gs). Signaling pathways mediated by A1 and A3 include adenyl cyclase inhibition, phospholipase C (PLC) activation and PI3K/Akt activation. A2A and A2B induce adenyl cyclase activity. ARs stimulate PI3K/Akt and the mitogen-activated protein kinase (MAPK) family which trigger specific cellular responses including the regulation of gene transcription. Abbreviations: ADP, adenosine diphosphate; AMP, adenosine monophosphate; Akt, serine-threonine protein kinase; ATP, adenosine triphosphate; CD39, cluster of differentiation 39; CD73, cluster of differentiation 73; CNT, Na^+^-dependent concentrative nucleoside transporter; ENT, Na^+^-independent equilibrative nucleoside transporter; PI3K, phosphoinositide 3-kinase, SAH, S-Adenosyl-L-homocysteine.

In the brain, adenosine can also be generated outside cells from ATP via sequential dephosphorylation reactions that are catalyzed mainly by nucleoside triphosphate diphosphohydrolase-1 (CD39), which transforms ATP into ADP and successively into AMP, and by the action of 5′-ectonucleotidase (CD73), which transforms AMP into adenosine. Then, extracellular produced adenosine can be transported inside cells by nucleoside transporters (ENTs or CNTs) or transformed into AMP through a phosphorylation reaction catalyzed by the adenosine kinase (AK) enzyme [64]. Alternatively, extracellular adenosine can be deaminated to inosine by adenosine deaminase (ADA). In the extracellular space, adenosine acts as a signaling molecule with different actions, including regulating the supply of oxygen on a demand basis, involving in immunomodulation, inducing repair and stimulating angiogenesis, and acting as a neuromodulator/neurotransmitter in the CNS [63,65] (Figure 4).

The cycle of adenosine being released and then uptaken, together with its extracellular metabolism, is conditioned on the state of the cells being in a healthy or pathological condition [66,67,68].

Adenosine acts through its “7-transmembrane G-protein coupled” purinergic P1 receptor subtypes, namely A1, A2A, A2B and A3, which are functionally linked to adenylyl cyclase (AC), to inhibit (A1R and A3R) or stimulate (A2AR and A2BR) the production of the second messenger cAMP. A2A and A2B receptors interact with Gs proteins to activate adenylate cyclase, while A1 and A3 interact with Gi/0 proteins, which reduce the activity of adenylate cyclase. Moreover, ARs can be involved in other cAMP-mediated pathways, including protein kinase C (PKC), phosphoinositide 3 kinase (PI3K), PI3K/Akt activation and mitogen-activated protein kinase (MAPK) pathways [12,14,69,70] (Figure 4).

It has been reported that under physiological conditions, the intra- and extracellular concentrations of adenosine in the brain are in the same range, between 20 and 30 nM, and that these concentrations are equilibrated by ENTs and CNTs, which, respectively, mediate the efflux and influx of adenosine across the cell membrane [18].

The action of adenosine through its receptors is different depending on the AR expression levels in specific tissues and the adenosine concentration. In fact, AR subtypes differ in their binding affinity to their natural ligand adenosine. A1R and A3R are high-affinity receptors that bind adenosine at concentrations of 0.1–3 nM, whereas A2AR is a high-affinity receptor that binds adenosine at 1–20 nM and A2BR is a low-affinity AR that is activated at an adenosine concentration higher than 1 μM. The receptors A1R and A2AR are widely expressed in the brain. Thus, under physiological conditions, adenosine signaling in the brain is mainly mediated via A1R and A2AR, whereas A2BR and A3R are more active in other body compartments where adenosine concentration is much higher compared to the brain [15,18,71,72]. It should be noted that the moderate expression of these adenosine receptors, as shown by glial cells under physiological conditions, is upregulated during CNS insults, such as neuroinflammation [73].

A1R and A2ARs are highly expressed in the brain. A1R is more abundant compared to A2AR, which exhibits a lower distribution under physiological conditions. A1R is expressed by neurons and is located presynaptically and postsynaptically. Adenosine acts as a neuromodulator that is involved in the control of transcellular messenger homeostasis and neurotransmitter release [14]. Moreover, adenosine is known to be a physiological neuromodulator that acts temporally at the place of production due to its short lifetime of around 10 s [74,75].

A1Rs and A2ARs are expressed by ECs, neurons, glial cells and immune cells [62,76]. Since ECs also express CD73, it is hypothesized that these cells are sensitive to the potential damage induced by extracellular ATP, which is known to be an injury signal in the brain [77,78], and might convert ATP into adenosine via the action of the ectoenzyme CD73. In this way, the resulting adenosine, acting locally through its receptor A1 and/or A2A, may modulate BBB permeability, with this effect being potentiated when both receptors are simultaneously activated. The literature shows evidence supporting AR signaling as a candidate for modifying endothelial barrier permeability [12,18,79,80].

Carman et al. extended on these studies by using an in vitro model of mouse brain endothelial cells (BEC), the Bend.3 cell line, an ex vivo model of mouse primary brain ECs, and in vivo mouse and rat models as well. The mouse Bend.3 cell line expresses A1R and A2AR mRNAs but does not express A2B and A3 receptors, whereas they express CD73. These authors demonstrated that the activation of A1R and A2AR by the selective agonists NECA (N-Ethylcarboxamidoadenosine) and Lexiscan (CVT-3146, also known as Regadenoson), respectively, induced a decrease in TEER, thus increasing the paracellular space between endothelial cells where TJs are located and, consequently, enhancing BBB permeability. These results suggest the involvement of the actin cytoskeleton in the maintenance of endothelial cell shape and barrier integrity, thus providing evidence for the involvement of adenosine signaling. The activation of A1Rs/A2aRs induced the formation of “actomyosin stress fibers”, which induced cytoskeleton changes in ECs that increased BBB permeability. As mentioned above, claudin-5, occludin and ZO-1 are TJ-associated proteins involved in the control of BBB structure and function. The treatment of Bend.3 cells with NECA or Lexiscan induced a decrease in the expression of claudin-5 and ZO-1 proteins, suggesting that the change in BBB permeability could be modulated by AR signaling through a change in TJ protein expression. Moreover, Western blot analysis of primary mouse BEC lysate showed the expression of A1Rs and A2ARs, thus suggesting that ECs of this in vitro model were capable of directly responding to adenosine [79].

On the other hand, Lexiscan or NECA administration (i.v.) to mice and rats increased the CNS entry of intravenously administered fluorescently labeled dextrans (10 kDa and 70 kDa); this effect was observed to be dose dependent, and the duration of this BBB permeability increase depended on the adenosine agonist’s lifetime, with the effect of NECA lasting longer (around hours) than that of Lexiscan (around minutes). The same effect was observed for macromolecules, such as antibodies, with this effect being significant for therapeutic agents that crossed the BBB. Moreover, immunofluorescent staining and fluorescent in situ hybridization methodologies applied to brain cortical slices demonstrated that ARs (A1 and A2A) co-stained with the CD31 endothelial marker [12]. From these in vivo results, it can be stated that adenosine, binding to its receptors, induces a transient and reversible BBB disruption that enhances BBB permeability.

In another study using an in vitro co-culture BBB model of a human cerebral microvascular endothelial cell line (hCMEC/D3) and a human normal glial cell line (HEB), it was demonstrated that the extract of Ginkgo biloba (EGb) induced a reversible increase in BBB permeability via the A1R signaling pathway. This effect was associated with an alteration in TJ ultrastructure, causing a reduction in the TEER parameter. In particular, the expression of phosphorylated TJ proteins, ezrin/radixin/moesin (ERM) and myosin light chain (MLC) (both ERM and MLC are actin-binding proteins) increased, whereas the expression of ZO-1, occludin and claudin-3 did not change. These observed effects were counteracted by the A1R antagonist DPCPX (1,3-dipropyl-8-cyclopentyl xanthine) [33].

All of the abovementioned results further suggest that adenosine, acting through A1Rs or A2ARs, could modulate BBB permeability to facilitate the entry of molecules into the CNS.

Other studies performed confirmed these results and expanded the investigation. Kim et al. demonstrated that BBB permeability could be controlled through adenosine signaling in a rapid, reversible and time-dependent way via endogenous mechanisms in which morphological changes in actin cytoskeletal reorganization, as well as claudin-5 and vascular endothelial (VE)-cadherin proteins, are involved. To perform their studies, these authors used the primary human brain microvascular ECs, HBMVECs, and the human EC cell line, hCMEC/D3. Both models highly express the ecto-enzymes CD73, CD39 and A2ARs. The authors demonstrated that the blockade of CD73 or A2AR inhibition hinders the migration of white blood cells into the CNS, whereas the activation of ARs by Lexiscan or NECA induces a decrease in the TEER parameter and, hence, an increase in BBB permeability. More specifically, the authors studied the kinetics of these effects and concluded that the duration of the increase in BBB permeability is proportional to the AR agonists’ lifetime, with this effect being reverted afterward. On the other hand, the authors described a rapid increase in cAMP after the treatment of HBMVECs with Lexiscan, which, at the same time, increases RhoA activity, a GTPase known to regulate cell structure and morphology through the re-organization of actin cytoskeletal proteins and the disruption of cell-to-cell junctions [12,80].

Moreover, it has been reported that the activation of ARs in mouse Bend.3 cells with the agonists Lexiscan and NECA induces a downregulation in the expression levels of claudin-5 and VE-cadherin, with Lexiscan’s effects being higher and faster than NECA’s effects. The A2AR specific antagonist, SCH58261, reverses NECA’s effects [12], demonstrating that the observed effects are mediated via A2AR. As already mentioned, claudin-5 is a TJ protein, whereas VE-cadherin is an adherens junction protein. Both proteins play a crucial role in maintaining endothelial barrier integrity.

Since A2BR and A3R are expressed at a lower level in the CNS compared to A1R and A2AR, there are not many reports on their role in the BBB. The A2BR agonist, BAY 60-656583, has been found to be involved in the regulation of cerebrovascular integrity in a rat transient middle cerebral artery occlusion model (tMCAO), inducing a decrease in tissue lesion volume and a decrease in the expression of TJ-associated proteins, such as ZO-1, thus protecting the BBB [81]. On the other hand, the A3R agonist, AST-004, has been found to play a neuroprotective role in a mouse model of traumatic brain injury (TBI). Mice treated with AST-004 showed less severe secondary brain injury (measured as cell death and loss of BBB breakdown) and a lower expression of neuroinflammatory markers compared to untreated mice. Moreover, the AST-004 treatment prevented the impairment of spatial memory in male mice [82]. More studies involving these two A2BRs and A3Rs should be performed.

Therefore, previous studies have identified a reversible paracellular increase in BBB permeability through AR signaling, which can be exploited in translational medicine to modulate the entry of molecules into the brain [12,50,62].

**Figure 5:**
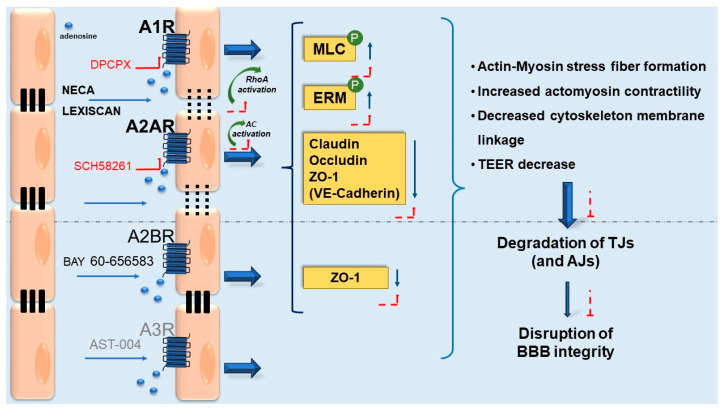
Adenosine modulation of BBB permeability proposed mechanisms. The activation of adenosine receptors after agonist binding induces specific signal transduction pathways depending on the AR subtype involved. The most expressed AR in brain microvascular endothelial cells (BECs) are A1 and A2A receptors, whereas A2B and A3 receptors are much less expressed. CD39 and CD73 are also expressed in BECs. Possible mechanisms of action mediated by ARs inducing the modification of BBB permeability are included in the figure and have been discussed in the text. In a proposed mechanism, agonists’ activation of ARs results in the activation of AC and RhoA-GTPase enzymes which induces the increase in phosphorylated MCL and ERM proteins, causing the activation of actomyosin contraction and subsequent BBB disruption. In another proposed mechanism, agonist activation of ARs induces alteration in the expression of TJ and AJs proteins (claudin, occludin, ZO-1, and VE-cadherin) and morphological changes in the actin cytoskeleton which induces a sequence of effects leading to BBB permeability alteration. In the same studies, ARs antagonists contrasted the effects caused by ARs activation. Abbreviations: AJs, adherens junctions; DPCPX (1,3-dipropyl-8-cyclopentyl xanthine; ERM, ezrin/radixin/moesin; MLC, myosin light chain; Neca, N-Ethylcarboxamidoadenosine; Lexiscan; TEER, transendothelial resistance parameter paracellular permeability; TJs, tight junctions; ZO-1, zona occludens-1.

A summary of the principal proposed mechanisms underlying adenosine modulation of BBB permeability acting through its receptors is shown in Figure 5.

### 4.2. Pathological Conditions

As already mentioned, the proper physiological function of the brain is guaranteed by regulating the transport of molecules into the brain (good molecules such as nutrients and bad molecules such as toxic metabolites), which is mediated by the BBB that plays a protective role for the CNS and maintains its homeostasis. In addition to its low permeability, the BBB endothelium is characterized by the presence of specific transport systems and drug efflux systems, including Pgp, MRPs and BCRP [80,83]. Therefore, the BBB has a friend role as well as a foe role, since it presents an obstacle for the entry of therapeutic substances into the brain, in particular in the treatment of neurological diseases and primary brain tumors where a limited penetration of drugs has been reported [84,85].

The BBB is a dynamic structure that is continually regulated by physiological and pathological factors. BBB dysfunction has been reported in many CNS pathological conditions, such as multiple sclerosis (MS) [86,87,88], hypoxic and ischemic insults, PD, AD [89,90], brain cancer [83] and inflammation [91]. Increased BBB permeability has also been described in normal aging [18,19,20]. BBB impairment in various pathological conditions has also been reported to be caused by structural changes due to the altered expression of TJ proteins. Moreover, the loss of TJ proteins has been associated with neuroinflammatory and neurodegenerative disorders [92,93]. Thus, the identification of the altered expression of proteins in the cerebral vascular endothelium during CNS pathological conditions may be used as a potential therapeutic target in the treatment of CNS diseases [18].

Much attention has been paid to how adenosine modulation of BBB permeability can be exploited to deliver drugs into the CNS to treat neurological diseases ranging from AD to brain tumors. To enable this, we need to first determine whether AR signaling regulates human BBB permeability in physiological and/or pathological conditions, and second, we need to understand the mechanisms that regulate brain endothelial barrier permeability and determine the role of AR signaling in the modulation of BBB permeability.

Several AR agonists and antagonists have been tested for their effects on BBB permeability, which differ in their affinity, time of action, and selectivity to ARs. For instance, the A2AR agonist, Lexiscan, is a low-affinity, short-acting and selective molecule, whereas CGS21680 is a high-affinity, selective molecule with a long duration of action and NECA is a nonselective agonist. In the case of low-affinity agonists, their potency of action depends on the AR expression levels in the targeted tissues and species, thus contributing to a higher tissue selectivity. Overall, previous studies have delineated the importance of receptor tissues and species differences, as these factors are related to drug responsivity, in an effort to optimize preclinical models that can be used for future clinical applications [83].

In some preclinical studies, the A2AR antagonist, caffeine, has been found to increase cognitive performance in AD animal models, demonstrating the effects of targeting A2AR in the therapy of AD [62,94,95]. Moreover, other clinical trials of neurodegenerative diseases, for instance, AD and PD, exploit the use of BBB-permeable adenosine-related compounds, mainly A2AR agonists and antagonists, alone or in combination with other drugs [96,97].

As has already been demonstrated, A2AR agonists increase BBB permeability via actin cytoskeletal reorganization, which is an integral part of the intercellular junction system. The proposed mechanisms to explain the modification in the functions of tight and adhesive junctions are (i) the increase in RhoA signaling activity and the consequent formation of actin stress fibers; (ii) the downregulation of VE-cadherin, ZO-1 and claudin-5 proteins; (iii) the reduced phosphorylation of adhesion-related factors; and (iv) the downregulation of P-glycoprotein expression in ECs. All of these alterations lead to an increase in the paracellular gap and an extended flow of substances across the BBB [18,80].

## 5. Adenosine Signaling under Pathological Conditions

An increase in the expression of A2AR in glial cells during hypoxia and inflammation has been described. In fact, A2AR agonists have been used to reduce inflammation during ischemic hemorrhagic shock to protect tissues from damage, mitigate inflammatory processes and protect neuronal cells [98,99].

Sleep restriction is another pathological condition in which increased BBB permeability and inflammatory responses have been described, with this effect being reverted after A2AR antagonism through SCH58261, which demonstrates the implication of A2AR in BBB dysfunction [100].

Gliomas are known to modify vascular permeability via BBB disruption. Consequently, to limit this pathological process, one tentative approach to repair the damage is by regulating certain proteins’ expression and function [101]. Interestingly, endothelial cells in cerebral vessels are characterized by the presence of A1R and A2AR. It has been shown that substances affecting these receptors regulate the permeability of the BBB. More specifically, it has been demonstrated that A2AR agonists increase BBB permeability, and the proposed mechanisms involve actin cytoskeletal reorganization at TJs [83]. Therefore, A2AR agonists may be used as potential therapeutic strategies in the co-treatment of brain cancer to increase the effectiveness of oncological therapy.

The A2AR agonist, Lexiscan, has been demonstrated to increase BBB permeability in in vivo models of mice and rats through the downregulation of TJ proteins and P-glycoprotein. However, Lexiscan is known to have a short circulating lifetime and show important systemic side effects, such as stroke, shortness of breath and nausea. Moreover, it has shown no efficacy in some clinical studies [80,83,94].

Interestingly, strategies to vehicle drugs into the brain, such as the application of NPs, have also been used in the treatment of brain tumors in preclinical studies; more specifically, NPs are conjugated with A2AR agonists, such as Lexiscan [102]. Compared to the use of A2AR agonists alone, Lexiscan-conjugated NPs had an enhanced selectivity and reduced systemic side effect, which improved targeted drug delivery to the CNS and prolonged its time of action upon opening the BBB [102,103]. Nevertheless, they showed some disadvantages such as potential neuro- and systemic toxicity, and only small-sized NPs (<12 nm) can cross the BBB. Thus, NP technology must be further studied [83].

It has been demonstrated that high concentrations of ATP are released during ischemia, inflammation or cell damage as a “danger signal”. Adenosine functions mainly as an anti-inflammatory and pro-resolving signal to suppress neuroinflammatory reaction and to reinforce the structural integrity of the CNS barriers [104]. ECs are capable of directly responding to extracellular adenosine. Since extracellular adenosine mediates many of its functions in inflammation or injury [105], we hypothesize that adenosine acting via ARs on ECs induces the recruitment of cells and/or molecules into the CNS to sites of damage or inflammation [62,91].

As already mentioned, conventional nanomedicine approaches take advance of ligands and NPs, whose physiochemical characteristics are employed to enhance drug delivery across the BBB. As a step up of this technology, some authors have created a targeted nanomedicine capable of bypassing the BBB and BCSFB to deliver specific A1R antagonists (theophylline and 1,3-dipropyl-8-cyclopentyl xanthine-DPCPX-) selectively to specific areas of the spinal cord by utilizing a retrograde neural tracer (wheat germ agglutinin-horseradish peroxidase, WGA-HRP). This targeted nanomedicine is able to revert the respiratory dysfunction caused by respiratory muscle paralysis during spinal cord injury (SCI) [106].

## 6. Conclusions

Despite its invaluable protective and functional role, the BBB is a major obstacle in the delivery of drugs for the treatment of CNS diseases.

The development of drugs for treating CNS diseases should be committed to both CNS drug discovery and CNS drug delivery.

Research has provided interesting outcomes in the development of new therapeutic strategies for effectively targeting a drug to the brain compartment, to minimize systemic side effects and to achieve the highest drug selectivity.

In this context, adenosine acting on distinct subtypes of P1 receptors expressed on BBB cells modulates the permeability of the BBB, as A1R and A2AR activation increases its permeability while A2BR and A3R stimulation maintains its integrity. It should be remarked that A1R and A2AR agonists, by briefly opening the BBB, allow drugs’ passage, thereby presenting a significant potential for the delivery of drugs to the CNS. Moreover, the use of appropriate selective AR antagonists could, in turn, limit the entry of harmful substances and inflammatory immune cells into the brain and contribute to the maintenance of CNS homeostasis. In addition, since ECs express ARs and the ectonucleotidases CD73 and CD79, signaling at ECs may represent a druggable endogenous mechanism for modulating BBB permeability to treat CNS diseases.

## Data Availability

Data sharing not applicable.

## Abbreviations

A1R, adenosine A1 receptor; A2AR, adenosine A2A receptor; A2BR, adenosine A2B receptor; A3R, adenosine A3 receptor; ABC, adenosine triphosphate (ATP)-binding cassette; AC, adenylyl cyclase; ABCs, arachnoid barrier cells; AD, Alzheimer’s disease; ADA, adenosine deaminase; ADP, adenosine diphosphate; AK, adenosine kinase; Akt, serine-threonine protein kinase; AMP, adenosine monophosphate; AMT, adsorptive-mediated transcytosis; AR, adenosine receptors; ATP, adenosine triphosphate; BBB, blood–brain barrier; BBBD, blood–brain barrier disruption; BCRP, breast cancer resistant protein; BCSFB, blood–cerebrospinal fluid barrier; BECs, brain endothelial cells; BM, basal membrane; CD31, cluster of differentiation 31; CD39, cluster differentiation 39 (ectonucleoside triphosphate diphosphohydrolase-1); CD73, cluster differentiation 73 (ecto-5′-nucleotidase); Cln, claudins; CMT, carrier-mediated transport; CNS, central nervous system; CNTs, concentrative nucleoside transporters; CPE, choroid plexus epithelial cells; CSF, cerebrospinal fluid; DBC, dural border cells; DHA, docosahexaenoic acid; CSF, cerebrospinal fluid; DPCPX, 1,3-dipropyl-8-cyclopentylxanthine; EBA, endothelial barrier antigen; E-cadherin, endothelial-cadherin; ECs, brain microvessel endothelial cells; EGb, extract Ginkgo biloba; ENTs, equilibrative nucleoside transporters; ERM, ezrin/radixin/moesin; GGTP, gamma-glutamyl-transpeptidase; Gi, inhibitory G protein; GLUT-1, glucose transporter-1; Go, other G protein; Gs, stimulatory G protein; GTP, guanosine triphosphate; HBMVECs, human brain microvascular endothelial cells; hCMEC/D3, human cerebral microvascular endothelial cell line; HEB, human normal glial cell line; IDDD, intrathecal drug delivery device; JAMs, Junction adhesion molecules; MAPK, mitogen-activated protein kinase; MFSD2a, major facilitator superfamily domain-containing protein 2a; MLC, myosin light chain; MRP, multidrug resistance-associated protein; MS, multiple sclerosis; NECA, N-Ethylcarboxamidoadenosine; NPs, nanoparticles; NVU, neurovascular unit; P1, Purinergic receptor type 1; PCs, pericytes; PD, Parkinson’s disease; PECAM-1, platelet endothelial cell adhesion molecule-1; Pgp, P-glycoprotein ; PI3K, phosphoinositide 3 kinase; PK, pharmacokinetics; PKC, protein kinase C; PLC, phospholipase C; RME, receptor-mediated endocytosis; RMT, receptor-mediated transcytosis; SAH, S-Adenosyl-L-homocysteine; SAS, subarachnoid space; TBI, traumatic brain injury; TJs, tight junctions; tMCAO, transient middle cerebral artery occlusion model; VE, vascular endothelial; vWf, von Willebrand factor; WGA-HRP, wheat germ agglutinin-horseradish peroxidase; ZO-1, zona occludens-1.
